# Photolithographic Fabrication of Micro Apertures in Dry Film Polymer Sheets for Channel Recordings in Planar Lipid Bilayers

**DOI:** 10.1007/s00232-019-00062-9

**Published:** 2019-03-12

**Authors:** Mario El Khoury, Tobias Winterstein, Wadim Weber, Viktor Stein, Helmut F. Schlaak, Gerhard Thiel

**Affiliations:** 1 Department of Electrical Engineering and Information Technology, Institute of Electromechanical Design, Microtechnology and Electromechanical Systems, TU Darmstadt, Darmstadt, Germany; 2 Membranbiophysik, Department of Biology, TU Darmstadt, Schnitspahnstrasse 3, 64287 Darmstadt, Germany; 3 Protein Engineering, Department of Biology, TU Darmstadt, Darmstadt, Germany

**Keywords:** Photolithography, Ion channel recording, Planar lipid bilayer

## Abstract

**Abstract:**

Planar lipid bilayers constitute a versatile method for measuring the activity of protein channels and pores on a single molecule level. Ongoing efforts attempt to tailor this method for detecting biomedically relevant target analytes or for high-throughput screening of drugs. To improve the mechanical stability of bilayer recordings, we use a thin-film epoxy resist ADEX as septum in free-standing vertical bilayers. Defined apertures with diameters between 30 µm and 100 µm were micro-fabricated by photolithography. The performance of these septa was tested by functional reconstitution of the K^+^ channel Kcv_NTS_ in lipid bilayers spanned over apertures in ADEX or Teflon films; the latter is conventionally used in bilayer recordings and serves as reference. We observe that the functional properties of the K^+^ channel are identical in both materials while ADEX provides no advantage in terms of capacitance and signal-to-noise ratio. In contrast to Teflon, however, ADEX enables long-term experimental recordings while the stability of the lipid bilayer is not compromised by pipetting solutions in and out of the recording chamber. Combined with the fact that the ADEX films can be cleaned with acetone, our results suggest that ADEX carries great potential for multiplexing bilayer chambers in robust and reusable sensing devices.

**Graphical Abstract:**

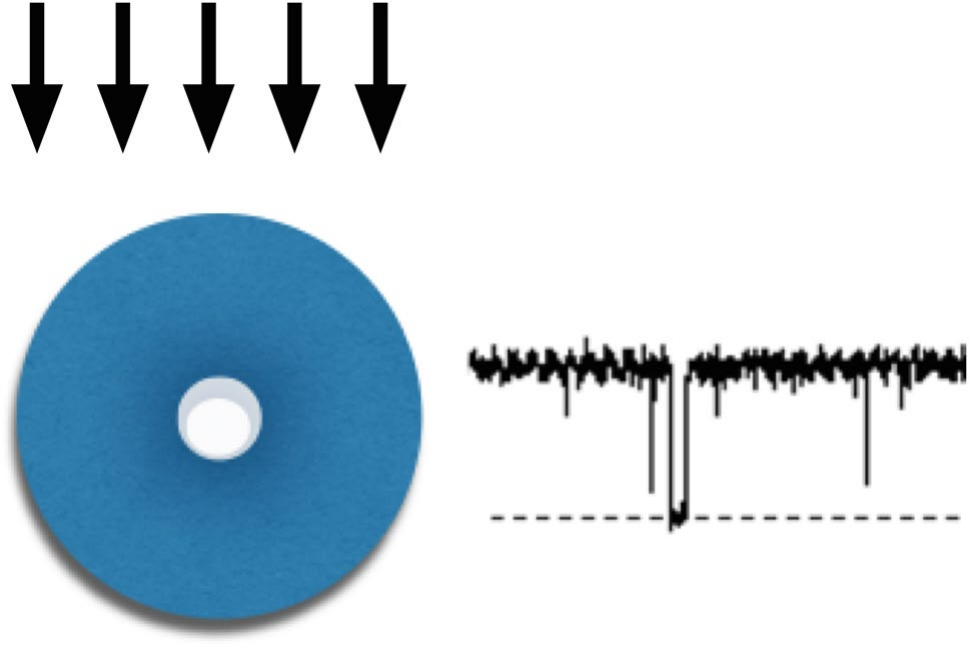

## Introduction

Channel proteins catalyze the diffusion of ions across lipid membranes. This can occur in a highly regulated and selective manner through canonical ion channels (Hille 2001), or less selectively *via* highly conductive beta-barreled pores (Delcour 2002). Considering the function of ion channels is crucial for a large variety of cellular events (Ashcroft 2006), their underlying structure/function relationships are intensively studied in the context of their physiological and pathophysiological activity (Kurachi and North 2004; Catterall et al. 2017). For the same reason, ion channels are also considered promising drug targets (Terstappen et al. 2010; Yu et al. 2016). In recent years, pore-forming proteins, so-called protein nanopores, have also received great attention from protein engineers (Ayub and Bayley 2016). These proteins can be genetically modified to sense various physical stimuli (e.g. voltage, mechanical stress) and chemical signals and molecules (e.g. ligands, pH, DNA etc.) in a highly sensitive and selective fashion. Sensing of chemical or physical cues is then translated into altered channel gating, which in turn affects the currents through the nanopores (Bayley and Cremer 2001). Because the unitary conductance of nanopores is generally high, conventional amplifiers can register any modulation of pore conductance and/or gating by the analyte of interest. The most prominent example of such a biotechnological application of conducting pores is the MinION device from Oxford Nanopore Technologies, which serves as a portable long-read DNA sequencing device (Fraiture et al. 2018).

Since ion channels and protein nanopores are membrane proteins, they have to be embedded in a lipid bilayer to be functionally analysed. There are many established electrophysiological techniques for measuring the activity of channels and nanopores in lipid bilayers in a cellular context or *in vitro* (Coronado and Latorre 1983; Hamill et al. 1981; Huxley 2002; Iwamoto and Oiki 2015; Lee et al. 2016; Hartel et al. 2018). Among these methods, the planar lipid bilayer (PLB) technique constitutes even 50 years after its invention (Müller et al. 1962; Montal and Müller 1972) one of the most versatile methods for monitoring the activity of single ion channels and protein nanopores as well as their sensitivity to chemical and physical queues (Zakharian 2013). Its key advantage over other electrophysiological approaches is that it allows recording of channel and pore activity on a true single-molecule level under very reduced and defined conditions e.g., phospholipid composition and electrolyte concentrations. All of these provide a range of experimental benefits. For example, it becomes possible to compare the functional features of a purified channel protein directly with the structural properties of the same isolated from crystallographic or cryo-EM structures. Another benefit of the bilayer technique relates to the fabrication of sensing devices. The literature reports multiple strategies for including miniaturized bilayer set-ups into portable sensing devices (Gu 2009; Gu and Shim 2010) or for multiplexing bilayers for high-throughput analysis (Prokofyev et al. 2014; Kawano et al. 2011). An inherent disadvantage of the PLB technique concerns the incorporation of channel and pore proteins without contaminations into the bilayer and the mechanic instability of the lipid bilayer membrane. To overcome these shortcomings, many attempts were made over the past decades: In a recent study, we reported a new technique which allows efficient synthesis and incorporation of ion channel proteins into planar lipid bilayers (Winterstein et al. 2018). This approach combines *in vitro* translation of ion channels into nanodiscs followed by their direct reconstitution from the micro scaffolds into planar lipid bilayers. This procedure turned out to be a fast, efficient and artifact-free method to reconstitute and measure ion channel activity *in vitro* (Winterstein et al. 2018).

A number of studies have also provided technical solutions for improving the stability of the classical bilayer system (Gu 2009; Tien et al. 1991; Mach et al. 2008; Kalsi et al. 2014). In conventional set-ups, the bilayer is formed over septa in Teflon, overhead transparency films (Winterstein et al. 2018; Bartsch et al. 2012). Also cuvettes of monolithic polystyrene with locally thinned walls are used (Williams1994). The apertures in the respective septa, which host the lipid bilayer, are produced by rather crude methods including mechanical punching (Heginbotham et al. 1999), electrical sparks (Bartsch et al. 2012) or by drilling (Williams 1994). The size of the resulting apertures, which are not under a good control of the experimenter, usually range between 50 µm and 200 µm in diameter (Williams 1994, Heginbotham et al. 1999, Bartsch et al. 2012). These systems are well suited for experimental work, yet highly sensitive to mechanical disturbances. In our experience, most bilayers that have been formed over a Teflon septum with apertures > 100 µm do not survive the exchange of a solution in one of the chambers by pipetting; this mechanic sensitivity generally confounds the routine characterization of channel functions and high throughput approaches. Another disadvantage associated with the large aperture concerns the high capacitance of the lipid bilayer membrane. The latter is a major source of noise in bilayer recordings and prevents the resolution of channel currents with small unitary currents or with high frequency gating (Hartel et. 2018).

Various micro-engineering techniques provide solutions to overcome the technical challenges associated with manufacturing small apertures of a well-defined size. This includes a range of mechanical and lithographic micro-fabrication approaches for producing small pores of defined size in a variety of materials (Kalsi et al. 2014; Pantoja et al. 2001; Castellana and Cremer 2006; Groves et al. 1997; Cheng et al. 2001; Fertig et al. 2001; Peterman et al. 2002; Baaken et al. 2008; Buchholz et al. 2008). The basic message of these studies is that smaller apertures reduce the mechanical sensitivity in conventional bilayer recordings indeed while increasing the signal-to-noise ratio. Yet, smaller pores may in some cases also prevent the incorporation of channel proteins into miniaturized bilayers (Pantoja et al. 2001).

In this work, we report a micromachining method for fabricating small and defined apertures in laminates of precast epoxy thin dry film photosensitive polymer sheet *ADEX* (DJ MicroLaminates, Inc). ADEX foils with defined apertures can be used as septa in conventional bilayer set-ups, where they exhibit excellent properties for bilayer formation and stability. We observe that the electrical activity of a model K^+^ channel is identical to recordings in Teflon foils, but resists mechanical perturbations that occur during buffer exchange. In addition, ADEX foils are chemically resistant to acetone. Therefore, the same septum can be cleaned and reused in subsequent recordings. This property and the ADEX foil photolithography-based fabrication method leads to a reusable well-defined aperture, which is accurately positioned on the foil surface. The chosen ADEX polymer is compatible with various microsystem fabrication and manufacturing techniques, where a mass production of ADEX foils can be realized. Therefore, these ADEX foils can be integrated into reusable Lab-on-chip devices enabling the recording of multiple bilayers. We assume that this technique can improve electrical recordings in reconstituted lipid bilayer membranes.

## Materials and Methods

### Preparation of Single Micropore Epoxy Films

**Fig. 1:**
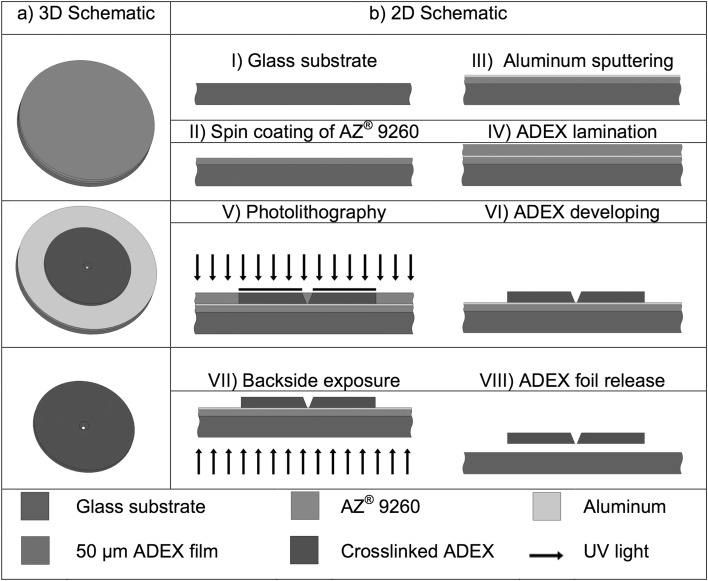
Microfabrication process for freestanding ADEX foils. (Step I) A borosilicate glass substrate (Schott BOROFLOAT® 33) is cleaned with acetone and isopropanol; (Step II) A photoresist layer of 7 µm AZ® 9260 was applied on the substrate; (Step III) 200 nm aluminum was sputtered on the photoresist; (Step IV) 50 µm ADEX layer was laminated on the aluminum; (Step V) Photolithography: Exposure to UV light and post exposure bake of ADEX; (VI) ADEX developing with PGMEA; (VII) Backside exposure of AZ® 9260; (VIII) ADEX foil is released: The aluminum is etched and the photoresist AZ® 9260 is developed with AZ® 400K

The single micropore ADEX foils used in this work are fabricated from a 50 µm photoimageable thin-film resist made of epoxy (ADEX TDFS A 50). They are prepared as follows (Fig. 1): A layer of 7-µm-thick photoresist (AZ® 9260) is deposited on a 100 mm borosilicate glass substrate (Schott BOROFLOAT® 33) and baked for 20 min at 100 °C on a flat hotplate. After a soft bake, a layer of 200 nm aluminum is sputtered on top of the wafer where it acts as a barrier between the AZ photoresist and the ADEX functional layer. The ADEX layer is laminated on the aluminum surface with a laminator (GBC HeatSeal ProSeries 3500LM) at a temperature of 75 °C and a lamination speed of 160 mm/min. The wafer is then baked for 5 min on a hot plate at 95 °C to eliminate material defects. To form the aperture foil, the ADEX polymer is in the next patterned by UV photolithography (200 mJ/cm^2^; wavelength: 365 nm). The exposed wafer is post-baked at 95 °C for 90 min with a heating and cooling ramp of 1 K/min. The ADEX is then developed with the SU-8 developer propylene glycol methyl ether acetate (PGMEA) for 10 min. To release the single-pore ADEX film, the backside of the wafer is exposed to UV light (800 mJ/cm^2^, wavelength: 436 nm); the sacrificial layer (AZ® 9260 + Aluminum) is then removed with the AZ® 400 K developer. The resist AZ® 9260 and the aluminum are removed in the same step. The ADEX foils are subsequently cleaned with isopropanol and deionized water. The procedure is schematically summarized in Fig. 1.

**Fig. 2:**
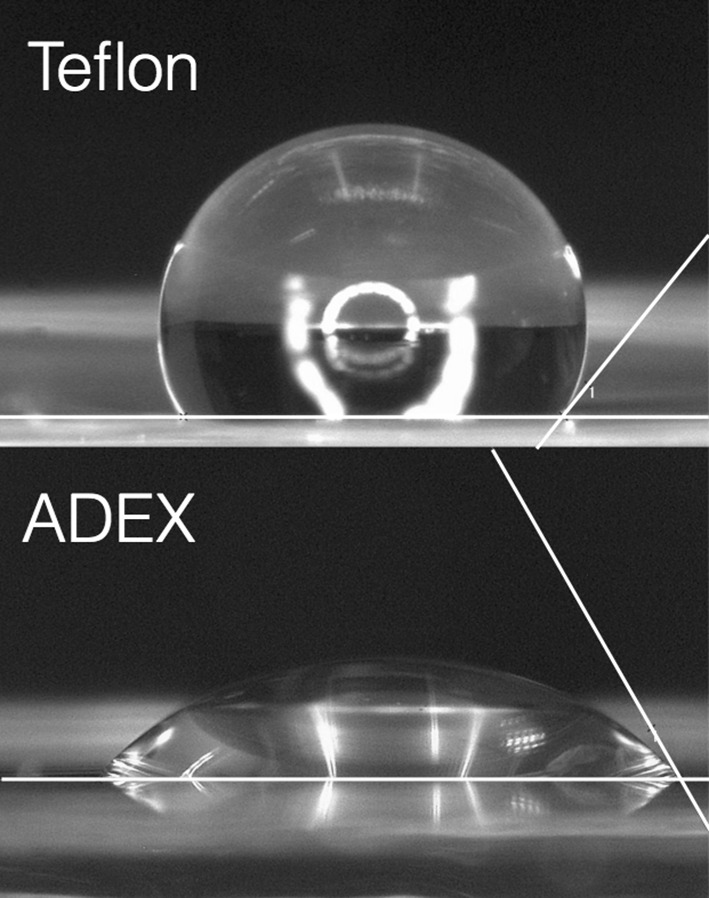
Hydrophobicity of ADEX versus Teflon foils. Contact angles of 10 µL water drops on foils of Teflon (top) or ADEX (bottom)

To estimate the hydrophobicity of the ADEX compared to conventional Teflon foils we measured the contact angle of a 10 µL drop of water on the respective materials. Exemplary images are shown in Fig. 2 revealing a mean contact angle of 130° on Teflon and 60° on ADEX foils. This demonstrates that Teflon is significantly more hydrophobic than ADEX.

### CLSM Measurements

Confocal laser scanning microscope (CLSM) measurements were performed on a LEICA TCS SP (Leica Microsystems, Mannheim, Germany) equipped with L20 x HCX APO 0.5 objective. For imaging of apertures in a foil, the latter was fixed with a thin agarose film support (1% in H_2_O) on the coverslips. The fluorescent probe fluorescein isothiocyanate (FITC, 0.1 mg/mL in 8% EtOH) was added on top of the foil from where it was sucked *via* the pore into the agarose bed. Images were taken with a 488 nm laser (emission range of 510 nm–530 nm) for FITC and a 633 nm laser (emission also 633 nm) for self-reflection of the foil surface. Analysis of the images was performed with Fiji—ImageJ (Schindelin et al. 2012).

### Protein Expression, Purification

*In vitro* expression of the K^+^ channel proteins was performed with the MembraneMax HN Protein Expression Kit (Invitrogen, Carlsbad, CA, USA) as described previously (Winterstein et al. 2018). The expression took place in the presence of different nanolipoproteins, so called nanodiscs (NDs). For the experiments described below, we used NDs provided with the commercial cell-free expression kit (MM, containing DMPC lipids) or MSP1D1-His discs ordered from Cube Biotech (Monheim, Germany). The latter were pre-assembled with DMPC, DMPG and POPC lipids. The scaffold proteins of all NDs were His-tagged to allow the purification of channel/ND-complexes *via* metal chelate affinity chromatography. The concentration of MSP1D1-His NDs in the reaction mixture was adjusted to 30 µM.

To purify the channel/ND-complexes, the crude reaction mixture was adjusted to 400 µL with equilibration buffer (10 mM imidazole, 300 mM KCl, 20 mM NaH_2_PO_4_, pH 7.4 with KOH) and subsequently loaded on an equilibrated 0.2 mL HisPur Ni–NTA spin column (Thermo Scientific). To allow the binding of the His-tagged NDs to the Ni–NTA resin, the columns were incubated for 45 min at room temperature and 200 rpm on an orbital shaker. Afterwards the buffer was removed by centrifugation. To eliminate unspecific binders, the column was washed three times with 400 µL of a 20 mM imidazole solution. Finally, the His-tagged NDs were eluted in three fractions with 200 µL of a 250 mM imidazole solution. All centrifugation steps were performed at 700 g for 2 min.

### Lipid Bilayer Experiments

Vertical lipid bilayer measurements were performed at RT (20–25 °C) in 1,2-diphytanoyl-*sn*-glycero-3-phosphocholine (DPhPC, Avanti Polar Lipids, Alabaster, AL, USA) membranes in symmetrical potassium chloride solutions (100 mM KCl, 10 mM Hepes, pH 7). The recording chambers were prepared as described previously (Braun et al. 2014a, b). In the present experiments, the 25-µm-thick Teflon septum was replaced by a 50-µm-thick ADEX foil, which was prepared as described before.

Lipid bilayers were formed inside the apertures in both types of septa by means of painting or pseudo-painting/air bubble technique (Müller et al. 1962; Braun et al. 2014b). To incorporate channel proteins into the lipid bilayer, a small amount (~ 2 µL) of the purified channel/ND-conjugates was added directly below the bilayer in the *trans* compartment with a bent 25 µL Hamilton syringe (Hamilton Company, Rene, NV, USA). For the reconstitution of single channel proteins, the first elution fraction was diluted with a 250 mM imidazole solution by a factor of 10^3^ to 10^6^ (Winterstein et al. 2018). After channel incorporation in the lipid bilayer, constant voltages between + 160 mV and − 160 mV were applied for 10 s to several minutes. Both compartments of the bilayer chamber were connected with Ag/AgCl electrodes to the headstage of a patch-clamp amplifier (L/M-EPC-7, List-Medical, Darmstadt, Germany). The electrode in the trans compartment served as ground. Currents were filtered with a 1 kHz 4-pole Bessel filter and digitized with a sampling frequency of 5 kHz by a 16-bit A/D-converter (LIH 1600, HEKA Elektronik, Lambrecht, Germany).

## Results and Discussion

**Fig. 3:**
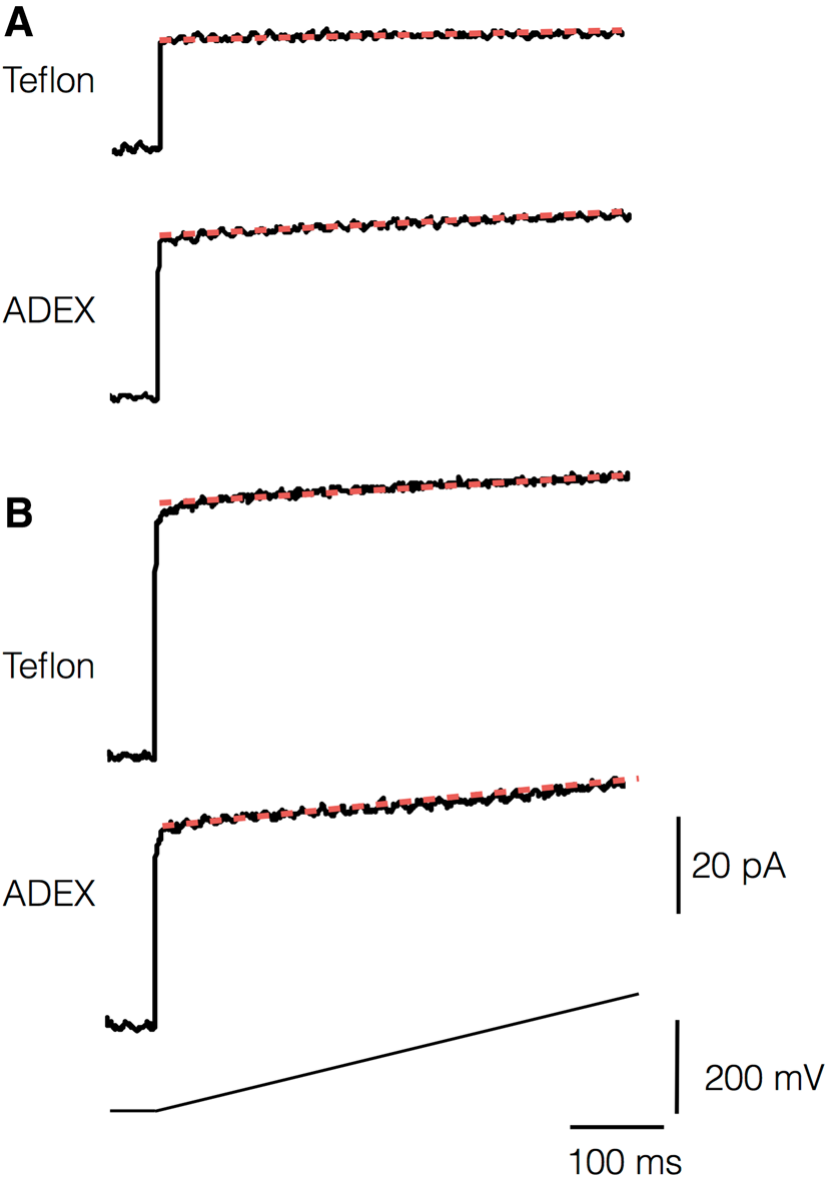
Capacitance and resistance of Teflon and ADEX foils. Intact Teflon or ADEX foils (**a**) or foils with approx. 100 µm large aperture (**b**) were sealed between two chambers of a planar lipid bilayer set-up. After filling both chambers with 100 mM KCl solution and after generating a stable DPhPC bilayer over the apertures in B, the current responses to a voltage ramp (lower trace) were measured. The initial current jump at the onset of the ramp is a measure for the capacitance. The resistance can be obtained from the slop (red line) of the current during the voltage ramp

Dry ADEX film sheets are suitable for fabricating complex apertures by photolithographic methods. To examine the application of these films as septum in planar lipid bilayer set-ups, we first measured the electrical properties of intact 50-µm-thick ADEX foils. The latter were, therefore, sealed with silicone grease between two chambers of a planar lipid bilayer set-up (Bartsch et al. 2012). After filling the chambers with 100 mM KCl, a voltage ramp was applied across the ADEX foil to measure its electrical properties in the bilayer set-up (Fig. 3). With *C*_c_ = *I*/(∆*V*/∆*t*) in which *I* is the initial current jump at the onset of the ramp and ∆*V*/∆*t* the slope of the voltage ramp we determine the capacitance *C*_c_ of the system. With intact ADEX foil in the bilayer set-up we obtained a value of 54 pF (54 ± 1.1 pF, *n* = 4) for (*C*_c_) From the slope of the subsequent current response, a resistance (*R*_c_) of 63 GΩ (mean 61 ± 3, *n* = 4) was estimated. The same experiments were performed with the usual 25-µm-thick Teflon foil, which is routinely employed for bilayer recordings (Winterstein et al. 2018; Sugawara and Hirano 2005). Based on measurements in 4 foils, we obtained a mean *C*_c_ value of 39 ± 1 pF and a mean *R*_c_ of 588 ± 179 GΩ. The results of these measurements show that, in the bilayer set-up, the ADEX foil has a lower resistance and approx. 1.5 times higher capacitance than a Teflon septum.

**Fig. 4:**
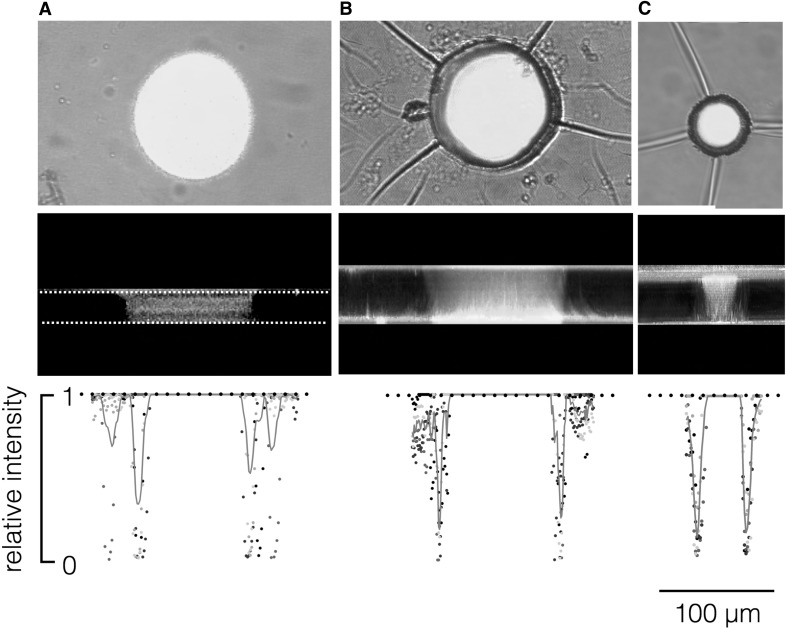
Imaging of apertures in Teflon and ADEX foils. The upper row shows a top view on single apertures in Teflon (**a**) and ADEX foils (**b, c**). Illustrated are overlays of bright field images (grey) with fluorescent images (green) from FITC in the pore. The central rows show the corresponding side view of the pores reconstructed from confocal scans of fluorescein fluorescence; the borders in the Teflon foil are indicated by dotted lines. The edges of the ADEX foil are visible from the self-reflection of the 633 nm laser, which does not occur in Teflon. The lower row reports data from individual intensity scans (grey points) and their mean values (red lines; *n* ≥ 4) for the respective pores. The data were obtained by measuring the grey value along a line at the equator of single pores in a top view perspective. Grey values from different pores were normalized to the same ordinate. Scale bar is valid for all panels

Next we generated, as illustrated in Fig. 1, cylindrical pores with different diameters in ADEX foils. To visualize the geometry of these pores, film sheets with a single aperture were incubated in a solution with FITC and imaged on a confocal microscope. For comparison, the aperture in Teflon films generated by electric sparks (Bartsch et al. 2012) was imaged in the same manner. Representative examples of top and side views of both preparations are shown in Fig. 4. The large and small apertures in the ADEX foil are perfectly spherical and highly reproducible. The standard deviation of the mean pore diameter is < 5% of the pore size based on 4 foils prepared in the same manner. In contrast, teflon-based apertures exhibit a larger variability around a mean diameter of 96.4 ± 14 µm; the standard deviation is as large as 15% of the pore size based on five foils prepared in the same manner.

The cross-section reveals that the apertures in ADEX foils have a slight cone shape in which the diameter on one side is approx. 10% smaller relative to the opposite rim. We can assign the larger pore diameter to the side of the film that was exposed to the UV light in the lithographic preparation.

To examine the application of ADEX septa in planar lipid bilayer (PLB) recordings, we used foils with apertures of approx. 100 µm diameter; for comparison, apertures with similar dimensions were also generated by electric sparks in Teflon (Fig. 4). Both septa were positioned in conventional cuvettes for a vertical bilayer set-up (Bartsch et al. 2012). Planar lipid bilayers of DPhPC phospholipid were then generated with the painting or pseudo-painting/air bubble technique (Müller et al. 1962; Braun et al. 2014b). After establishing a stable bilayer, the capacitance (*C*_p_) and resistance (*R*_p_) of single pores were measured in both systems from voltage ramps (Fig. 3). In case of ADEX septa, the *C*_p_ value was 57 pF (mean 57 ± 1 pF, *n* = 4) (Fig. 3b) in a pore with a mean diameter of 102 ± 2 µm. To obtain the specific capacitance of the lipid bilayer (*C*_m_) in the pore, the mean *C*_c_ value measured in Fig. 3a was subtracted from *C*_p_ and the remaining value normalized to the mean area of the pore. In this way, we can estimate a mean-specific membrane capacitance *C*_m_ of 0.3 ± 0.01 µF/cm^2^ and 0.6 ± 0.02 µF/cm^2^ for bilayers in pores of approx. 100 µm diameter in ADEX and Teflon foils, respectively. The *C*_m_ value in Teflon is compatible with data reported for other bilayer systems including Teflon as septum (Winterstein et al. 2018, Sugawara and Hirano 2005). The specific capacitance of the membrane in the ADEX foil is lower. We cannot provide a full explanation for this phenomenon. But based on systematic considerations of bilayer properties (White 1985) we must assume that the very hydrophobic nature of Teflon causes a higher degree of solvent extrusion from the bilayer than the less hydrophobic ADEX material (Fig. 2). This will eventually result in a higher content of solvent, which remains in the bilayer in the ADEX foil and explains their lower specific capacitance (Dilger and Benz 1985).

The results of these experiments indicate that the apertures in ADEX films carry both disadvantages (e.g., lower resistance) and advantages (e.g., lower specific capacitance) compared to similarly sized apertures in conventional Teflon films. ADEX septa are thus generally suited to host stable, free-standing planar lipid bilayers. Yet, because of inferior electrical properties, ADEX films will not necessarily improve the signal-to-noise ratio in ion channel recordings compared to Teflon foils.

**Fig. 5:**
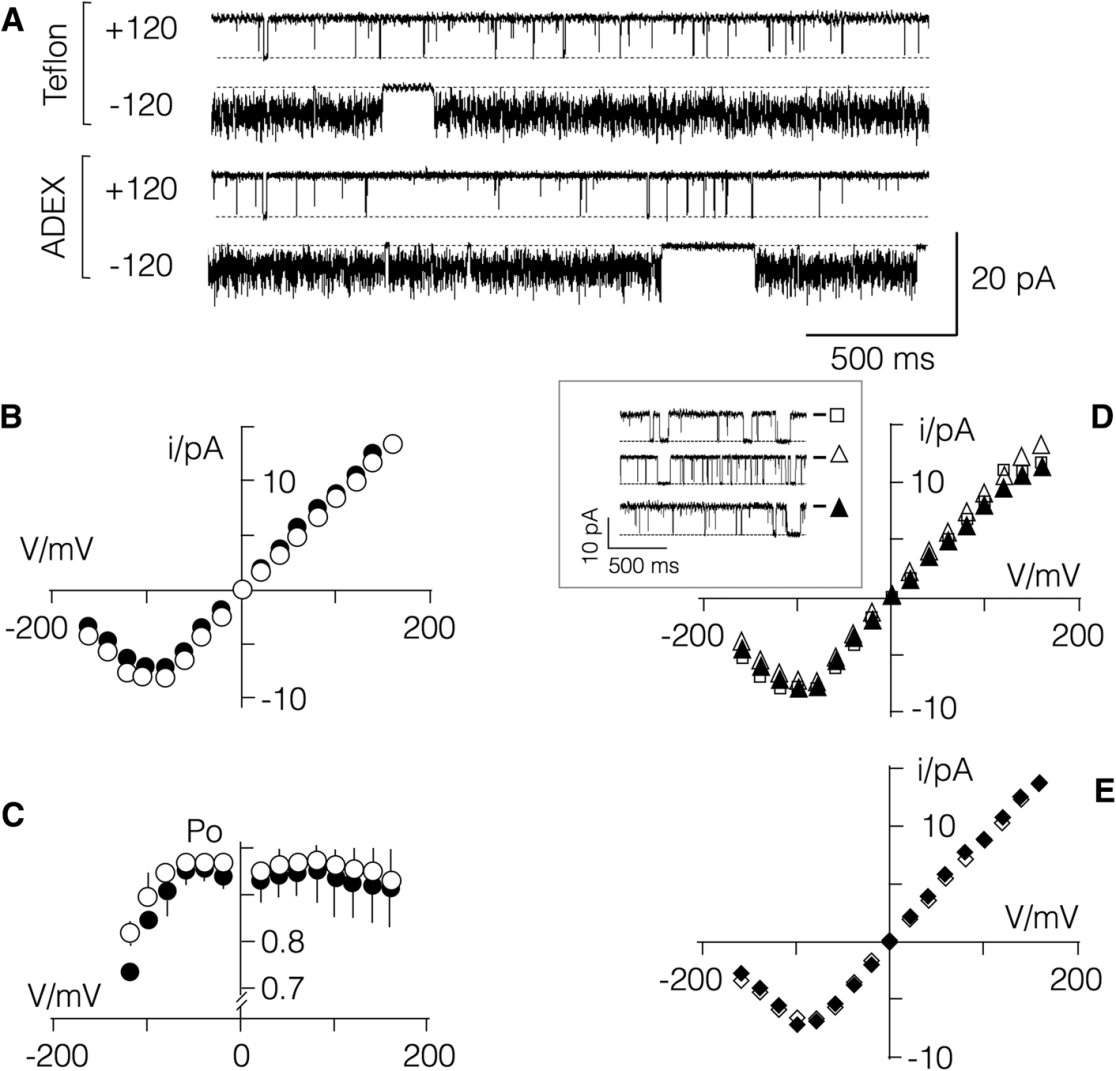
Comparative recordings of channel activity in septa from Teflon and ADEX films. (**a**) Typical channel fluctuations of Kcv_NTS_ channel at ± 120 mV in symmetrical solution with 100 mM KCl. Data were measured in DPhPC bilayer painted over an aperture in Teflon (open circles) or ADEX (closed circles) foils with apertures of 100 µm and 50 µm, respectively. (**b**) Mean *I*/*V* relation (± SD) of unitary Kcv_NTS_ currents from recordings as in (**a**) in Teflon (black circles; *n* = 4) or in ADEX foil (open circles; *n* = 3). **c** Mean open probability/voltage (Po/V) relation (mean ± SD) of Kcv_NTS_ channel from recordings as in (**b**). **d** I/V relations of Kcv_NTS_ channel reconstituted in bilayers in ADEX foils as in (**a**) with apertures of 100 (unfilled square) 50 (unfilled triangle) or 30 (filled triangle) µm diameter; *inset*: representative current traces at − 120 mV for three different apertures in ADEX foil. **e***I*/*V* relations of Kcv_NTS_ channel in ADEX foil with 100 µm diameter as in A before (filled diamond) and after (unfilled diamond) cleaning septum in Acetone

In subsequent experiments, we compared the functional properties of a reference K^+^ channel, the small viral protein Kcv_NTS_ (Rauh et al. 2018), that has been reconstituted in lipid bilayers formed over ADEX- and Teflon-based septa. Representative recordings of single channel fluctuations in a DPhPC bilayer generated over an approx. 100-µm-large hole in Teflon foil or over approx. 50-µm-large hole in ADEX foil are shown in Fig. 5. In both cases, we measured the same type of channel activity. At positive voltages, the channel exhibits well-resolved channel openings and closings. At negative voltages of approx. − 100 mV, the unitary openings become increasingly noisy; the latter is caused by a typical fast gating at negative voltages, which cannot be resolved in conventional recording set-up (Rauh et al. 2018). From the unitary channel fluctuations, we constructed the current/voltage relation as well as the open probability/voltage in both recording conditions (Fig. 5b, c). A comparison of the data shows that the basic functional features of the K^+^ channel can be measured in both recording set-ups and independently of the size of the aperture in the ADEX septum (Fig. 5b, d). The expected higher content of solvent, which remains in bilayers in ADEX septa, has no apparent impact on the functional features of the model channel. Notably this channel is very small (Braun et al. 2014a) and should hence be sensitive to parameters, which strongly affect the physicochemical features of the bilayer. It is also worth mentioning that the insertion of a channel into a bilayer formed over the ADEX septum occurs with the same bias as for a Teflon septum (Winterstein et al. 2018).

**Fig. 6:**
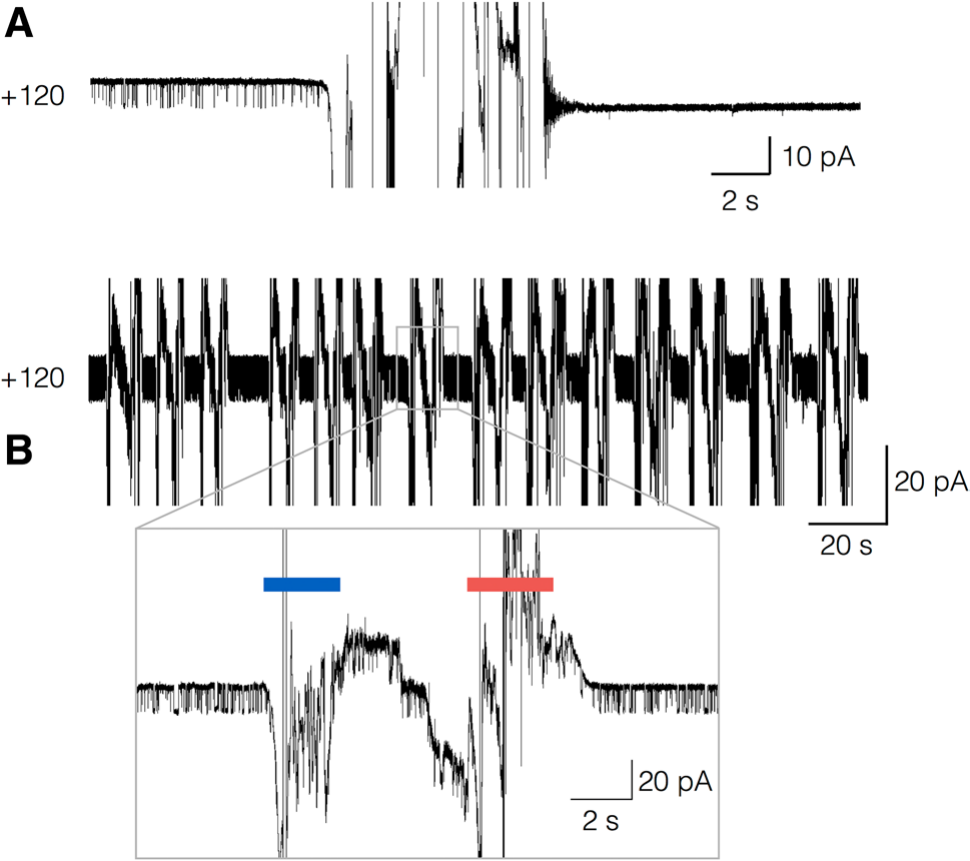
Sensitivity of bilayers in Teflon and ADEX septa to solution exchange. Continuous recordings of KcvNTS channel activity at + 120 mV in symmetrical solution with 100 mM KCl in Teflon (**a**) and ADEX (**b**) foil. During the recording, the solution of the trans-chamber was removed and resupplied from a 5000 µL pipette. This procedure resulted in A in a destruction and reformation of the bilayer. While the original bilayer (left) exhibited an active channel the reformed bilayer (right) did not. The same procedure could be frequently repeated with the ADEX septum (**b**) without loosing the bilayer with the active channel. One cycle of removing (blue bar) and resupplying of the solution (red bar) in B is magnified

To test the stability of recordings in ADEX-based septa, measurements as in Fig. 5 were kept for as long as 48 h before they were actively terminated. In all cases, the recordings proceeded without experiencing any instability of the bilayer. This suggests that bilayers formed over ADEX septa are very stable. In a next set of experiments, we further examined the stability of channel recordings in ADEX- and Teflon septa. To this end, we periodically removed the medium in the *trans* chamber of the bilayer set-up. This operation generally destroys the bilayer over a Teflon septum (not shown). In other cases, the bilayer is first destroyed and then spontaneously reforming during the refilling of the measuring chamber. Since this newly formed bilayer does no longer contain the channel of interest (Fig. 6a) the procedure does not fulfill the purpose of a solution exchange.

The situation is very different for lipid bilayers formed over a septum in ADEX foil; the representative example in Fig. 6b shows that the chamber could be frequently emptied and refilled without compromising the quality of the bilayer. The results of these experiments demonstrate that lipid bilayer membranes formed over ADEX septa are mechanically much more stable compared to those in Teflon.

ADEX films are also resistant to chloroform and acetone, which provides the possibility of cleaning them from lipids and proteins. To test the possibility of reusing ADEX septa for bilayer recordings, we measured channel activity as in Fig. 5 in an ADEX film with a 30 µm pore. The septum was then washed for 1 min with acetone, for 5 min with isopropanol and subsequently rinsed twice in distilled water. After painting a new bilayer over the aperture, the same type of K^+^ channel was reconstituted and measured. The functional data, which are here represented by the unitary I/V relation in Fig. 5e, show no difference between the channel performance in fresh or recycled septum. The results of these experiments show that ADEX foils can be easily reused after cleaning with acetone for channel recordings, and provides a critical property for the design and handling of sensor devices based on ADEX foils.

## Conclusion

In conclusion, we find that 50-µm-thick ADEX foils provide a suitable material for micro-machining small and reproducible apertures by lithography. They can host stable membranes in conventional free-standing vertical planar lipid bilayers set-ups. Comparative analysis of channel activity reveals that the basic functional features of a model K^+^ channel are indistinguishable between recordings in a conventional septum from Teflon or in ADEX films with different size apertures. The epoxy-based septa show no particular electrical advantage, which would improve the signal-to-noise ratio in channel recordings. But the material guarantees long-lasting measurements of channel activity and allows an easy exchange of the buffer solution in the recording chambers without compromising the stability of the bilayer. This stability of bilayers in ADEX septa together with the resistance of the material to acetone for cleaning and the quasi-unlimited possibility of fabricating complex apertures suggests that this material provides a promising basis for multiplexing septa in devices for high-throughput bilayer recordings.
